# Prosurvival autophagy is regulated by protein kinase CK1 alpha in multiple myeloma

**DOI:** 10.1038/s41420-019-0179-1

**Published:** 2019-05-21

**Authors:** Marilena Carrino, Laura Quotti Tubi, Anna Fregnani, Sara Canovas Nunes, Gregorio Barilà, Livio Trentin, Renato Zambello, Gianpietro Semenzato, Sabrina Manni, Francesco Piazza

**Affiliations:** 1 Department of Medicine, Hematology and Clinical Immunology Branch, University of Padova, Padova, Italy; 2 Veneto Institute of Molecular Medicine (VIMM), Padova, Italy; 3 Boston Children’s Hospital/Harvard Medical School, Boston, MA USA

**Keywords:** Myeloma, Drug development, Macroautophagy

## Abstract

Multiple myeloma (MM) is a tumor of plasma cells (PCs). Due to the intense immunoglobulin secretion, PCs are prone to endoplasmic reticulum stress and activate several stress-managing pathways, including autophagy. Indeed, autophagy deregulation is maladaptive for MM cells, resulting in cell death. CK1α, a pro-survival kinase in MM, has recently been involved as a regulator of the autophagic flux and of the transcriptional competence of the autophagy-related transcription factor FOXO3a in several cancers. In this study, we investigated the role of CK1α in autophagy in MM. To study the autophagic flux we generated clones of MM cell lines expressing the mCherry-eGFP-LC3B fusion protein. We observed that CK1 inhibition with the chemical ATP-competitive CK1 α/δ inhibitor D4476 resulted in an impaired autophagic flux, likely due to an alteration of lysosomes acidification. However, D4476 caused the accumulation of the transcription factor FOXO3a in the nucleus, and this was paralleled by the upregulation of mRNA coding for autophagic genes. Surprisingly, silencing of CK1α by RNA interference triggered the autophagic flux. However, FOXO3a did not shuttle into the nucleus and the transcription of autophagy-related FOXO3a-dependent genes was not observed. Thus, while the chemical inhibition with the dual CK1α/δ inhibitor D4476 induced cell death as a consequence of an accumulation of ineffective autophagic vesicles, on the opposite, CK1α silencing, although it also determined apoptosis, triggered a full activation of the early autophagic flux, which was then not supported by the upregulation of autophagic genes. Taken together, our results indicate that the family of CK1 kinases may profoundly influence MM cells survival also through the modulation of the autophagic pathway.

## Introduction

Multiple myeloma (MM) is a tumor of plasma cells (PCs) that accumulate in the bone marrow (BM) causing BM insufficiency, osteolytic bone lesions and hypercalcemia^1^. MM cells massively secrete immunoglobulins, provoking blood hyperviscosity, renal insufficiency and amyloidosis^2^, and causing endoplasmic reticulum (ER) stress in the cell^3^. To manage it, the ubiquitin-proteasome system, the unfolded protein response and the autophagic pathway cooperate to avoid proteotoxicity.

Autophagy is a process of self-degradation of cellular components, such as long-lived proteins, portion of cytoplasm and damage organelles^4^. In MM, silencing of autophagic molecules, such as ATG7 and SQSTM1/p62, induces cells death^5^. Compounds such as 3-methyladenine (3-MA) that prevents autophagy at the earliest stage of autophagosome formation, and chloroquine that disrupts lysosomal acidification preventing autophagosomes fusion and degradation, produce cytotoxic effects to MM cells^6^. However, also the hyperactivation of autophagy is maladaptive for MM cells. Caspase-10 protects from excessive autophagy, indeed its downmodulation causes autophagic cell death without hallmarks of apoptosis^7^. Moreover, Rapamycin (an mTOR inhibitor that increases the autophagic flux) exerts anti-MM effects in preclinical studies^6^. Therefore, autophagy must be finely regulated in MM, since both its inhibition and its hyperactivation culminate in cell death.

Recently, protein kinase CK1α (hereafter referred as CK1α) was described as a novel regulator of the autophagic pathway in RAS-driven cancers^8^. CK1α, encoded by the *CSNK1A1* gene, is the smallest isoform of the CK1 family, which is composed by 7 members^9^. CK1α regulates the subcellular localization of the transcription factor FOXO3a, which transcribes autophagy-related genes. The AKT-mediated phosphorylation of S315 of FOXO3a, together with the subsequent CK1α-dependent phosphorylations of S318/321, prompts FOXO3a nuclear exclusion^8^. CK1α also downregulates the autophagic flux in colon cancer^8^, osteosarcoma and neuroglioma^10^. Moreover, CK1α regulates several molecular pathways, involved in MM pathobiology^9,11^. Others and we have recently demonstrated that CK1α inactivation results in MM cell death^12,13^, pointing to a role for CK1α in growth, survival and proliferation of malignant PCs. Its inhibition in association with anti-MM drugs (such as bortezomib and lenalidomide), synergistically empowers their efficacy^13^.

Since the autophagic pathway and apoptotic cell death are strongly interconnected^14,15^, here we investigated a potential intertwining between autophagy and CK1α inactivation in controlling MM cell death. To this aim, we inhibited the members of the CK1 family CK1α and CK1δ with the chemical D4476, a cell-permeant inhibitor of CK1α and δ isoforms^16^ and, to specifically test the role of α isoform, we silenced CK1α through RNA interference (RNAi).

Unexpectedly, we found that the two approaches to inactivate CK1α had different consequences on autophagy. Indeed, D4476 treatment impaired the autophagic flux after lysosome fusion, while CK1α silencing did not promote the nuclear localization and the transcriptional activity of FOXO3a, with the final result of de-fueling the autophagic process.

Since both D4476 treatment and CK1α silencing culminate in MM cell death^13^, our findings suggest that the deregulation of autophagy upon CK1α inactivation may be deleterious for MM cells, pointing to a role for this kinase as a master regulator of stress signaling in malignant PCs.

## Results

### CK1α inactivation affects LC3B cleavage and p62 expression

**Fig. 1:**
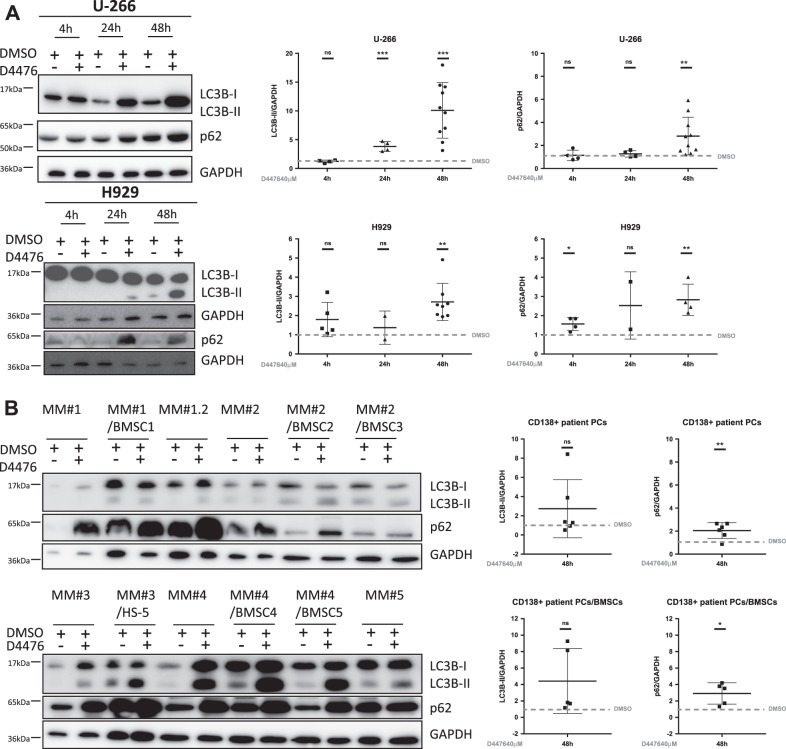
CK1α inhibition determines LC3B-II and p62 accumulation. **a** U-266 (upper panel) and H929 (lower panel) cells were treated with D4476 40 μM for different time points (4–24–48 h). **b** Different MM patient-derived PCs (MM#1/1.2, MM#2, MM#3, MM#4, MM#5. MM#1, and MM#1.2 refer to PCs collected from the same patient in two different stages of the disease) cultured alone or with stromal cells (BMSC1, BMSC2, BMSC3, BMSC4 or HS-5) were treated with D4476 40 μM for 48 h. LC3B and p62 expression was evaluated by WB (left panel). GAPDH was used as loading control. Densitometric values for each time points (right panels) are reported as mean ± SD over DMSO treated cells (collected at the same time point) (dashed lines) of at least 4 (4 h), 4 (24 h) and 10 (48 h) independent experiments for U-266; 4 (4 h), 2 (24 h) and 4 (48 h) independent experiments for H929; 6 independent CD138+ patient PCs cultured alone and 5 independent PCs co-cultured with BMSCs/HS-5, being the densitometric value of D4476 treatment always reported over the densitometric value of DMSO of the same time point. Non parametric *t*-test was applied. *Indicates *p* < 0.05, **indicates *p* < 0.01, ***indicates *p* < 0.001, ns indicates no relevant variation compared to the control population

**Fig. 2:**
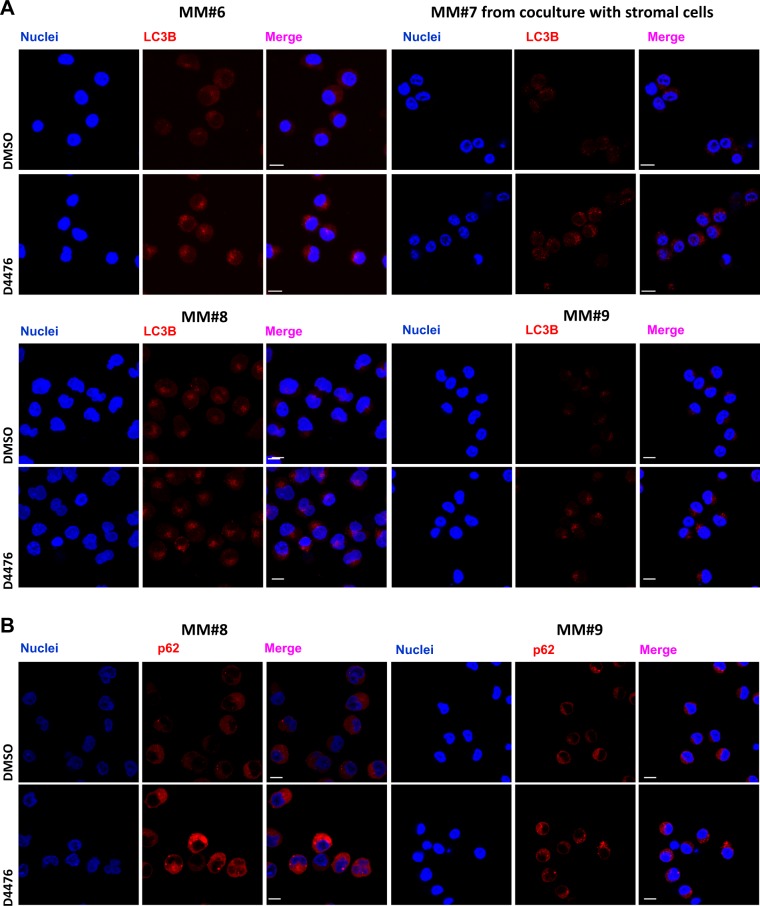
LC3B and p62 expression and localization in D4476 treated-MM patient-derived PCs. Immunofluorescence staining of LC3B (**a**) and p62 (**b**) (red) in CD138+ patient-derived PCs (MM#6, MM#8, and MM#9) cultured alone or in the presence of HS-5 stromal cells (MM#7) treated with D4476 40 μM for 48 h. Nuclei are stained with DAPI (blue). For all the images 63x oil objective was used. Scale bar: 10 μm

Since both normal^17^ and malignant^5^ PCs require autophagy for survival, CK1α regulates autophagy in RAS-driven cancer^8^ and CK1α downmodulation enhances the autophagic flux in osteosarcoma and neuroglioma cells^10^, we evaluated the effects of CK1α inactivation on autophagy in MM. Upon autophagy activation, LC3B is cleaved to form the cytosolic LC3B-I, which is lipidated to form LC3B-II, that is incorporated in autophagosomes^18^. LC3B-II interacts with p62, a cargo degraded together with LC3B-II in the autophagic vesicle. Treatment of RAS wt cells U-266 and NRAS-G12D mutated H929 cell lines for 4–24–48 h with D4476 40 µM revealed a modulation of LC3B-II and p62 expression in both cell lines. Densitometric analysis showed an increase of both autophagic markers expression mainly at 48 h of treatment (Fig. 1a). We also treated purified PCs from five MM patients, cultured alone or co-cultured with stromal HS-5 or patients derived bone marrow stromal cells (BMSC), with D4476 40 μM for 48 h. We observed a substantial increase of p62, but not of LC3B-II, in most of the MM patient PCs even in growing condition with protective stromal cells (Fig. 1b). The level of LC3B-II is variably affected by D4476 treatment in MM patient plasma cells. Table 1 reports the clinical features of MM patients analyzed. Next, we assessed the consequences of CK1α silencing on p62 and LC3B levels. We employed either H929 MM cells transiently transfected with siRNA targeting *CSNK1A1* or a stably transduced H929 cell clone bearing an IPTG-inducible *CSNK1A1*-directed shRNA (hereafter named H929 shRNA 6044). Analysis of CK1α expression at different times (24–48–72 h after siRNA electroporation or 24–48 h-7 days of 1 mM IPTG treatment) confirmed a significant reduction of the protein (Fig. S1a, b). LC3B-II and p62 expression was highly variable in CK1α-silenced cells as compared to controls (H929 electroporated with a scramble (SCR) siRNA and H929 shRNA 6044 not treated (NT) with IPTG) (Fig. S1a, b). Nevertheless, immunofluorescence analysis of LC3B and of p62 in patient-derived PCs (cultured alone or with HS-5 stromal cells) and in U-266 and H929, clearly revealed that CK1α/δ inhibition with D4476 for 48 h increased autophagosomes formation compared with DMSO treated controls (Fig. 2 and S2). We speculated that the observed accumulation of both LC3B-II and p62 in D4476-treated cells could depend on different mechanisms: (i) induction by the autophagic stimuli of new p62 synthesis, replacing the p62 protein being degraded inside the autophagosomes; (ii) blockage of the autophagic flux without consequent degradation of the autophagosomes. To test which one of these mechanisms could account for our findings, we analyzed p62 mRNA expression. We cultured H929 in FBS free medium (as positive control of autophagy), finding, as expected, an increase in p62 mRNA expression (Fig. S3a). Treatment of different MM cell lines with D4476 also caused an increase of p62 mRNA (Fig. S3b). On the contrary, in H929 shRNA 6044 treated with IPTG, the transcription of p62 mRNA was poorly reduced, while no changes were observed in H929 expressing a scramble sequence (H929 shRNA SCR) treated with IPTG (Fig. S3c). A modest increase in p62 mRNA was observed in H929 electroporated with siRNA against *CSNK1A1*, while no changes were observed in U-266 (Fig. S3d). Thus, chemical inhibition of CK1α/δ or CK1α level reduction with RNAi may affect in a different way the transcription of p62. As the NF-ĸB pathway was shown to transcribe p62 mRNA^19^, we evaluated if its activity would be increased upon CK1α inhibition, leading to enhanced p62 transcription. Indeed, an increase of Ser 536 residue phosphorylation of p65 was present after D4476 treatment, suggesting that NF-ĸB pathway may be triggered (not shown). The NF-ĸB pathway was analyzed also upon CK1α silencing producing not informative results (not shown).

**Table 1:** Clinical and pathological features of MM patients analyzed

Sample (MM PCs)	%intramedullary PCs	Paraprotein type	ISS	ISS-R	High LDH	Karyotype	C	R	A	B
(A)										
MM1	80%	IgG/κ	III	III	Y	t(4;14), gain 1q, hypodiploid	N	N	Y	N
MM1.2	75%	IgG/κ			Y	t(4;14), gain 1q	N	N	Y	Y
MM2	70%	IgG/κ+κ	III	II	N	Normal	Y	Y	Y	Y
MM3	100%	IgG/κ	II	II	N	Normal	N	N	Y	Y
MM4	30%	IgG/λ	III	II	N	Normal	N	N	Y	N
MM5	100%	κ	ND	ND	N	Normal	N	N	Y	Y
MM6	70%	IgG/λ	II	II	N	Hyperdiploid	N	N	Y	Y
MM7	70%	IgA/κ	II	II	N	Hyperdiploid	N	N	Y	N
MM8	60-70%	IgG/λ	III	III	Y	del17p, gain1q	N	Y	Y	N
MM9	95%	IgG/κ	I	I	N	t(11;14)	N	N	N	Y
(B)										
BMSC1	50%	IgG/λ	I	I	N	Hyperdiploid	Y	N	Y	Y
BMSC2	100%	IgA/κ	III	II	N	del1p, hypodiploid	Y	Y	Y	Y
BMSC3	30%	κ	NA	NA	ND	gain1q				
BMSC4	45%	IgG/κ	II	II	N	Hyperdiploid	N	N	N	Y
BMSC5	70%	IgA/λ	I	I	N	Hyperdiploid	N	N	Y	Y

MM plasma cells (A) and MM BMSC (B) were isolated from the bone marrow of patients. Clinical staging was performed according to the International Staging System (ISS; albumin levels ≤ or > 35 g/L; β2-microglobulin levels ≤ or > 3.5 mg/L) and to the International Staging System-Revisited in 2015 (ISS-R, in addition to the ISS criteria, considered the presence of high level of LDH and the presence of chromosomal abnormalities). The presence of high lactate dehydrogenase LDH was evaluated. All features are reported as data at diagnosis

*PC* plasma cell, *BMSC* bone marrow stromal cell, *ISS* International Staging System, *ISS-R* International Staging System-Revised, *LDH* lactate dehydrogenase, *C* calcium elevated, *R* renal failure, *A* anemia, *B* bone disease, *Y* yes/present, *N* no/absent, *NA* not available, *ND* not determined

Different results obtained with D4476 and CK1α silencing could be associated with a compensation mechanism performed by other CK1 isoforms in CK1α silenced cells. However, RT-qPCR analysis of all the CK1 isoforms demonstrated the specific downregulation of only the CK1α isoform (Fig. S4).

### A different modulation of the autophagic flux is observed upon CK1 inhibition and CK1α silencing

**Fig. 3:**
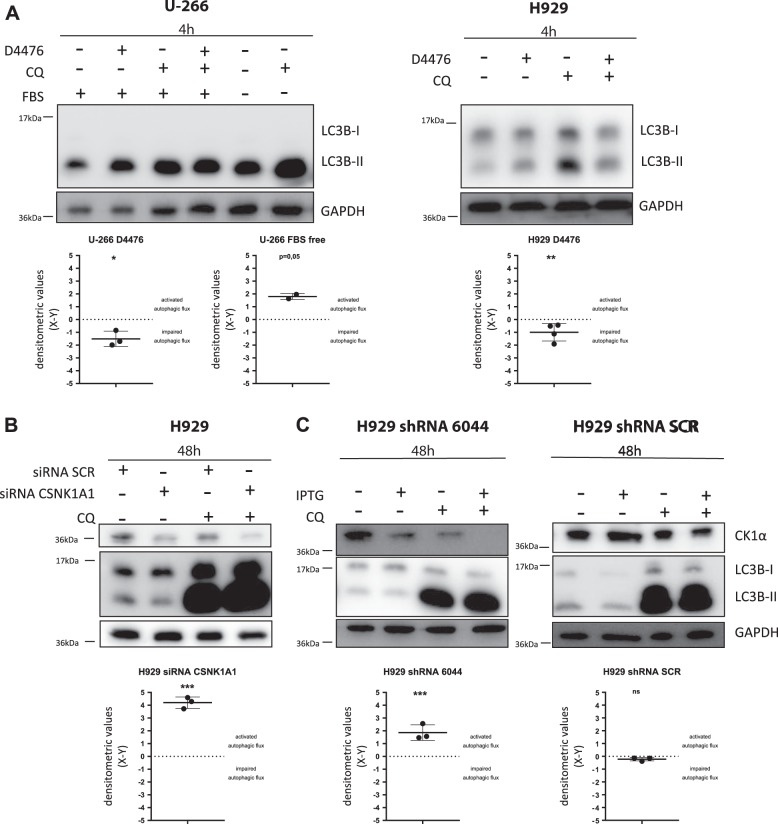
The autophagic flux is differently regulated by CK1 inhibition with D4476 and by CK1α silencing. Representative WB of LC3 turnover assay. **a** U-266 and H929 cell lines treated with the CK1α/δ inhibitor D4476 40 µM, chloroquine (CQ) 40 µM or the combination of the two compounds for 4 h. U-266 cells were also cultured in serum-free media for 4 h in the presence or absence of CQ 40 µM. **b** H929 transfected with SCR siRNA or *CSNK1A1*-directed siRNA and collected after 48 h in the presence or absence of CQ 40 µM. **c** H929 shRNA 6044 (left) and H929 shRNA SCR (right) clones treated with IPTG 1 mM for 48 h cultured in the presence or absence of CQ 40 μM. The upper part of each panel shows a representative WB of LC3B and CK1α expression upon treatments. GAPDH was used as loading control. The lower part of each panel shows the LC3 turnover assays, in which the densitometric quantification of LC3B-II bands was expressed as X = (treatment + CQ)-(treatment) and Y = (CQ)-(untreated cells). X > Y indicates activation of the autophagic flux (consequently X−Y > 0). Data are represented as the mean ± SD of at least 3 independent experiments. Non parametric *t*-test was applied. *Indicates *p* < 0.05; **indicates *p* < 0.01; ***indicates *p* < 0.001; ns indicates no relevant variation between X and Y values

Next, we evaluated autophagic flux induction by performing LC3 turnover assay. We used chloroquine (CQ) to block autophagic vesicles degradation in combination with CK1α inactivation, evaluating LC3B-II expression. We treated U-266 and H929 with D4476, CQ or with the combination of the two for 4 h, finding that D4476 treatment impaired the flux. As positive control of autophagy induction, we cultured U-266 in serum-free media for 4 h and we treated these cells with CQ, finding that the flux was properly induced (Fig. 3a). LC3 turnover assay performed after 48 h of D4476 treatment, demonstrated an impairment of the flux also at this time point(Fig. S5). This assay was also employed in H929 silenced with *CSNK1A1* siRNA (Fig. 3b) and in the IPTG-treated H929 shRNA 6044 cells (Fig. 3c). At variance with the D4476, we found an induction of the flux. As expected, no alteration of the flux was observed in H929 shRNA SCR treated with IPTG (Fig. 3c).

**Fig. 4:**
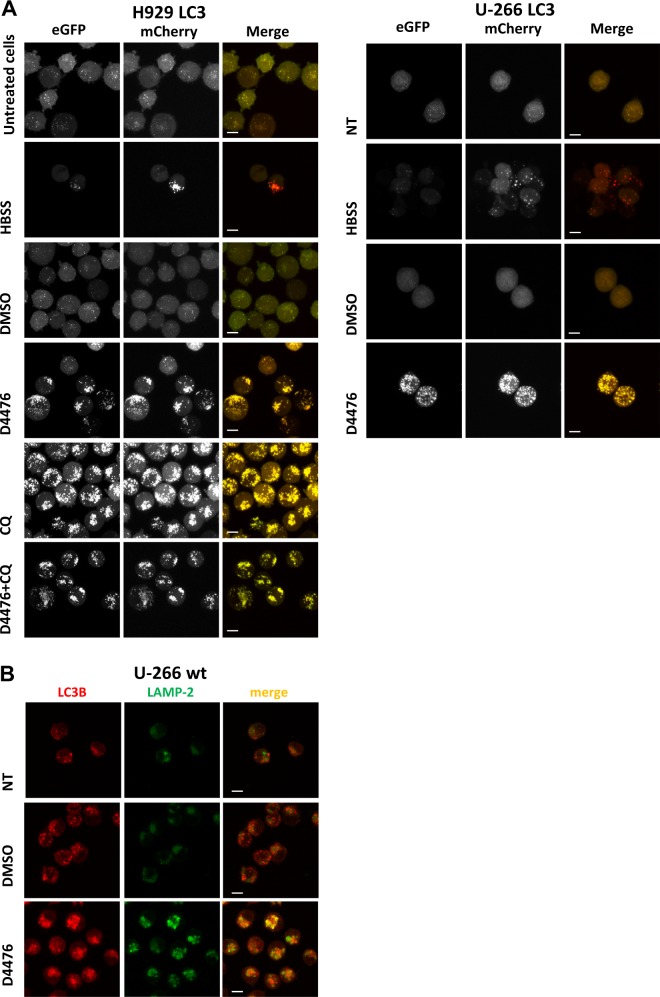
CK1α/δ chemical inhibition with D4476 blocks the autophagic flux after autophagosome-lysosome fusion. **a** H929 cells expressing mCherry-eGFP-LC3B protein (H929 LC3 clone, left panel) and and U-266 cells expressing mCherry-eGFP-LC3B protein (U-266 LC3, right panel) starved with HBSS, treated with DMSO or with D4476 40 μM for 48 h. H929 LC3 were also treated with CQ 40 μM alone or in combination with D4476 40 μM for 48 h. Yellow fluorescence resulting by the emission of both mCherry (red) and eGFP (green) indicates a blockage of the autophagic flux. Red fluorescence resulting by the quenching of eGFP fluorescence indicates a correct induction of autophagic flux. **b** Immunofluorescence staining of LC3B (red) and LAMP2 (green) in U-266 cell line treated with D4476 40 μM for 48 h. Nuclei are stained with DAPI (blue). For all the images 63x oil objective was used. Scale bar: 10 μm

To confirm these opposite results, we generated MM clones expressing LC3B tagged with both mCherry (pH insensitive sensor) and eGFP (pH-sensitive sensor). Upon autophagosomes formation, both tags emit light, resulting in yellow fluorescence, but after their fusion with the lysosome, the acid pH inside the lysosome quenches the green fluorescence (eGFP), resulting in red autolysosomes. Red vesicles formed when double tagged LC3B-expressing H929 and U-266 cells (named H929 LC3 and U-266 LC3) were serum-starved in HBSS. Importantly, treatment with both D4476, CQ and the combination of the two treatments, induced yellow vesicles formation confirming an impairment of the autophagic flux (Fig. 4a). Treatment with the vehicle DMSO did not cause autophagosomes formation (Fig. 4a). D4476 induced yellow vesicles accumulation also at shorter time points (18–24h) (Fig. S6). We next asked if D4476 could compromise autophagosomes transport to lysosomes or could induce an alteration of lysosomes acidification. We stained autophagosomes with an antibody against LC3B, and lysosomes with one against the lysosome surface protein LAMP-2. A partial colocalization of the two proteins suggested that in D4476-treated cells, autophagosomes correctly fused with lysosomes, therefore another cause of the lack of GFP fluorescence shutting off is likely to occur, perhaps affecting the acidification of the lysosomes (Fig. 4b).

**Fig. 5:**
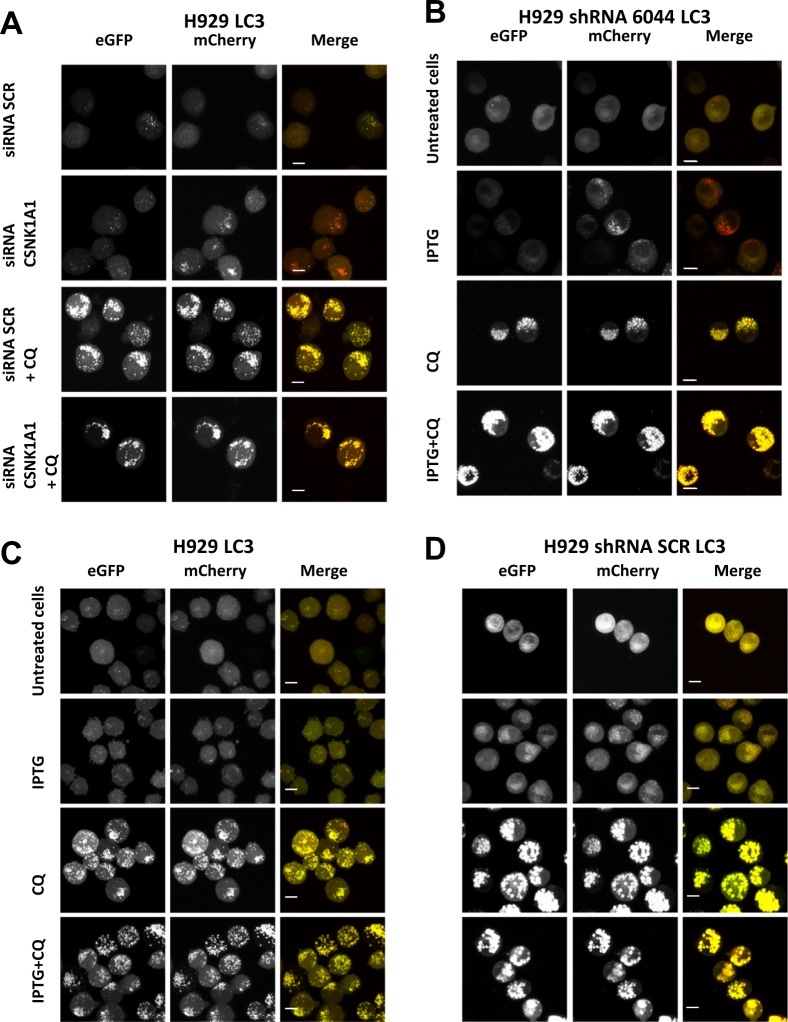
The autophagic flux is activated upon CK1α silencing. **a** H929 LC3 transfected with SCR siRNA and *CSNK1A1*-directed siRNA were treated with CQ 40 μM and collected after 48 h; **b** H929 shRNA 6044 LC3, **c** H929 LC3 and **d** H929 shRNA SCR LC3 were treated with IPTG 1 mM, CQ 40 μM or with the combination of the two for 48 h. Yellow fluorescence indicates a blockage in autophagic flux. Red fluorescence indicates a correct induction of autophagic flux. For all the images ×63 oil objective was used. Scale bar: 10 μm

We next evaluated the autophagic flux upon RNAi of the *CSNK1A1* gene. We electroporated *CSNK1A1* and SCR siRNAs in the H929 LC3 clone and we treated cells with CQ. CK1α silencing induced the formation of red autolysosomes and CQ treatment, as expected, was associated with the accumulation of yellow vesicles (Fig. 5a). Importantly, also IPTG treatment of H929 LC3 shRNA 6044 (Fig. 5b), but not of H929 LC3 (Fig. 5c) and of H929 LC3 shRNA SCR (Fig. 5d) induced red autolysosomes formation, confirming that upon CK1α silencing the flux is correctly induced. Treatment with CQ alone or in combination with IPTG determined yellow autophagosomes accumulation (Fig. 5b–d).

Altogether, these results indicate that in MM cells the autophagic flux is impaired by the CK1α/δ inhibitor D4476, while the specific knockdown of the CK1α isoform is associated to the unleashing of autophagy at least till the formation of acidic autolysosomes.

### The transcription factor FOXO3a localizes in the nucleus upon CK1α/δ inhibition but not upon CK1α-silencing

**Fig. 6:**
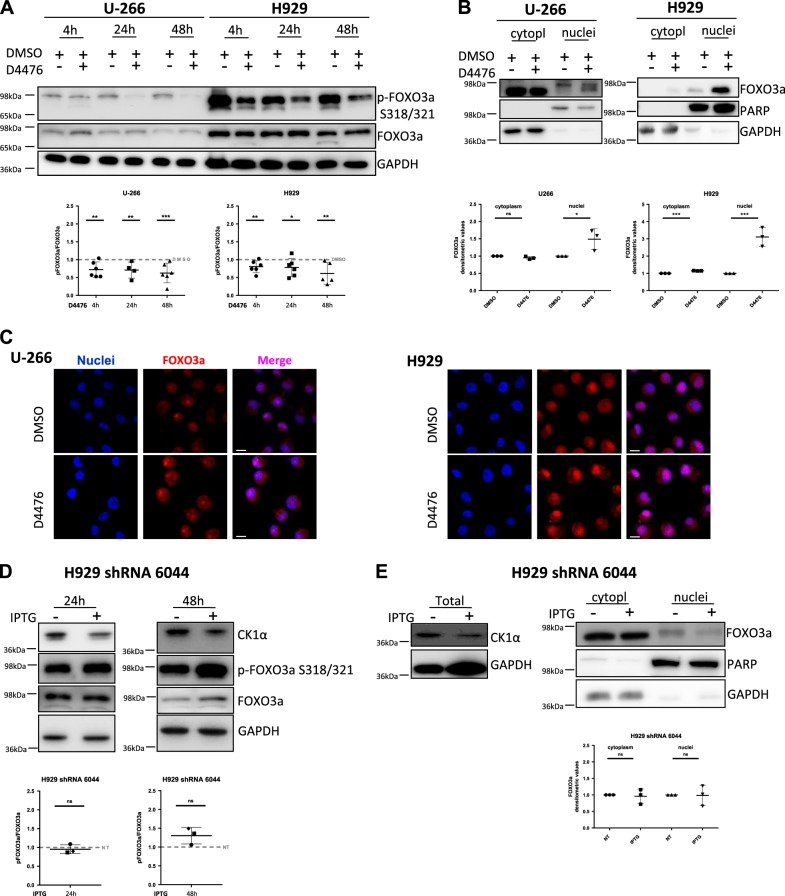
FOXO3a transcription factor localized in the nuclei in CK1α/δ chemically inhibited MM cells, but not in CK1α-silenced MM cells. **a** WB of S318/321 phosphorylation of FOXO3a (p-FOXO3a S318/321) and total FOXO3a expression in U-266 and H929 treated with DMSO or D4476 40 μM for 4 –24–48 h (upper panel) and densitometric analysis (lower panel). GAPDH was used as loading control. Densitometric values are reported as mean ± SD over DMSO treated cells (dashed lines) of at least 7 (4 h), 5 (24 h) and 7 (48 h) independent experiments for U-266; 7 (4 h), 7 (24 h) and 6 (48 h) independent experiments for H929. **b** WB of FOXO3a expression after cytoplasmic and nuclear proteins fractionation in U-266 and H929 treated with D4476 40 μM for 48 h (upper panels) and correspondent densitometric values of FOXO3a expression normalized over GAPDH (for cytoplasm) and over PARP (for nuclei) (lower panel). Densitometric values are reported as mean ± SD of 3 independent experiments for each cell line. **c** Immunofluorescence staining of FOXO3a (red) in U-266 and H929 treated with D4476 40 µM for 48 h. Nuclei are stained with DAPI (blue). For all the images ×63 oil objective was used. Scale bar: 10 μm. **d** WB of CK1α, of Ser318/321 phosphorylation of FOXO3a (p-FOXO3a S318/321) and of total FOXO3a in H929 shRNA 6044 treated with IPTG 1 mM for 24–48 h (upper panel) and correspondent densitometric analysis (lower panels). Densitometric values are reported as mean ± SD over untreated cells (NT, dashed lines) of at least 3 independent experiments for each time points; GAPDH was used as loading control. **e** CK1α and FOXO3a expression in total protein lysates and nuclear/cytoplasmic fractionation in H929 shRNA 6044 treated with IPTG 1 mM for 48 h. GAPDH was used as loading control for total and cytoplasmic lysates, while PARP for nuclear lysates. Densitometric values of FOXO3a normalized over GAPDH (for cytoplasm) and over PARP (for nuclei) are reported as mean ± SD of at least 3 independent experiments. Non parametric *t*-test was applied. *Indicates *p* < 0.05; **indicates *p* < 0.01; ***indicates *p* < 0.001; ns indicates no relevant variation compared to control population

FOXO3a transcribes autophagy-related genes and, when phosphorylated, translocates in the cytoplasm, becoming inactive. The AKT-dependent phosphorylation of S351 of FOXO3a is involved in nuclear-cytoplasmic shuttling and primes the subsequent CK1α dependent phosphorylations of S318-321^8^. We firstly treated U-266 and H929 cells with D4476, finding a reduction of S318-321 phosphorylations (Fig. 6a). Next, we performed nuclear and cytoplasmic proteins fractionation and WB analysis of FOXO3a expression in U-266 and H929 treated with D4476, finding that FOXO3a mainly localized in the nucleus (Fig. 6b). Immunofluorescence analysis of FOXO3a confirmed this finding (Fig. 6c).

Different results were obtained upon CK1α silencing. In IPTG-treated H929 shRNA 6044, the silencing of CK1α did not lead to the reduction of S318-321 FOXO3a phosphorylations (Fig. 6d) and FOXO3a did not accumulate in the nuclei (Fig. 6e). This latter result was also confirmed in CK1α-silenced U-266 shRNA 6044 clone (not shown).

### A transcriptional program that sustains the autophagic flux is induced upon CK1α/δ inhibition, but not upon CK1α silencing

To deepen these findings, we next studied the expression of some autophagy-related genes. In the positive control of autophagy induction, i.e. H929 cells cultured in serum-free media, upregulation of the transcription of all autophagy-related genes evaluated was observed, as opposed to cells cultured in standard conditions (Fig. S7a). Importantly, we found an upregulation of the same genes also in cells treated with D4476, even if with some differences in the cell models analyzed, being more pronounced in U-266 and INA-6 (Fig. S7b–d). Differently, a clear induction of an autophagic transcriptional program was not observed upon CK1α silencing with only some genes being weakly modulated (Fig. S7e–g). As expected, no increase in autophagic gene transcription was observed in H929 shRNA SCR treated with IPTG (Fig. S7h).

We next asked if the different results obtained with inhibition or silencing of the kinase could be due to a residual presence of the kinase in the CK1α silenced cells.

Therefore, we prolonged the IPTG treatment of H929 shRNA 6044 up to 7 days in order to further reduce CK1α expression. We did not observe any reduction of FOXO3a phosphorylation nor an increased transcription of autophagic genes in IPTG-treated cells despite a clear further downregulation of CK1α (Fig. S8).

To additionally reduce CK1α protein we treated CK1α-silenced cells with Lenalidomide 10 μM, which induces the proteasomal degradation of CK1α^13,20^. Although Lenalidomide strongly enhanced the reduction of CK1α, no alterations of FOXO3a phosphorylation were observed (not shown), suggesting that in MM FOXO3a phosphorylation could not depend on CK1α.

### Dual silencing of CK1α and CK1δ does not affect pro-autophagic gene expression

Since D4476 is a double CK1α/CK1δ inhibitor, we asked if the combined silencing of both kinases could yield the same results of D4476 on autophagy. To this aim, we electroporated a siRNA targeting CK1δ mRNA (*CSNK1D*) or a SCR siRNA in H929 shRNA 6044 and we cultured electroporated cells in the presence or absence of IPTG in order to silence CK1α. We first evaluated if CK1δ silencing could induce apoptosis of MM cells, observing a mild but statistically significant increase in apoptotic cells (not shown). Next, we analyzed FOXO3a phosphorylation, finding that the silencing of CK1δ or CK1α or both did not reduce S318-321 phosphorylations of FOXO3 (Fig. S9a, b). Transcription of autophagic genes was not induced in CK1δ-, CK1α- and CK1α/δ-silenced MM cells (with the exception of *LC3B* whose expression was reduced in CK1δ silenced cells), suggesting that in these conditions FOXO3a likely does not localize in the nucleus (Fig. S9c).

## Discussion

Recently, we demonstrated that CK1α supports MM growth by impinging on several survival signaling cascades and that CK1α inactivation synergistically cooperates with bortezomib and lenalidomide in inducing MM cell death^13^. Based on the discovery of CK1α role in the regulation of the autophagic pathway in RAS-driven cancer^8^, and being autophagy a fundamental survival stress-managing pathway for PCs, we investigated the role of CK1α on autophagy in MM.

Treatment of MM cell lines and patient-derived PCs with D4476 led to an accumulation of the autophagic markers LC3B-II and p62 in the cell lines and an increase in p62 in patient-derived PCs. LC3B-II increase was not statistically significant in MM-derived PCs, probably due to the small sample size analysed (Fig. 1). Moreover, CK1α inhibition in MM caused autophagic vesicles accumulation in both RAS wt (U-266) and RAS-mutated (H929) cells (Fig. S2). To note, U-266 are IL-6 secreting cells and this cytokine is known to activate the RAS pathway^21^. At variance with these findings, upon CK1α silencing an increased expression of the two autophagic markers was not observed (Fig. S1). The results obtained with D4476 were different from the ones obtained with the silencing of CK1α and were not coherent with previously published literature regarding CK1α inactivation in other cancers^8^. In particular, the increased expression of the cargo p62 suggests an impairment in the autophagic flux upon D4476 treatment. However, if the autophagic stimulus induces massive p62 transcription and translation, an accumulation of p62 could still be observed even in a context of correctly activated autophagic flux^22^. The increased p62 mRNA levels observed in D4476 could be associated to an elevated activity of NF-ĸB, as demonstrated by the D4476 mediated upregulation of Ser 536 phosphorylation of p65. The effects of CK1α inactivation on the NF-ĸB pathway were analyzed in activated B cell-like diffuse large B-cell lymphoma (ABC-DLBCL)^23^, demonstrating that CK1α inactivation resulted in downregulation of the NF-ĸB pathway, activated downstream the B-cell receptor (BCR) signaling. The BCR is lost in PCs, therefore the activation of NF-ĸB pathway could rely on different stimuli. Further analysis of the NF-ĸB activating pathways would be needed to clarify why an activation of NF-ĸB instead of a downregulation, was observed in D4476 treated cells. Analysis of p62 transcription produced different outcomes with the two silencing techniques, suggesting that the weak changes observed, have probably a statistical, but not a biological significance.

The fact that the autophagic flux resulted blocked upon D4476 and activated after CK1α silencing (Fig. 3a–c and S5), evidenced also using the pH-sensitive MM clones expressing mCherry-eGFP-LC3B protein (Figs. 4a–5 and S6), raises the question of the specificity of D4476, which is a dual inhibitor of CK1α and CK1δ, and of the role of this last as compared to CK1α on autophagy. The first point was addressed in the original work describing the inhibitor^16^ and, even if most of the kinases tested were unaffected by D4476, there remains the possibility of an off-target effect. The second point was addressed in the present paper. CK1δ inactivation, even if it caused a mild apoptotic effect in MM cells, did not result in a substantial modification of the autophagic pathway. Most importantly, the double silencing of CK1α and CK1δ led to the same results as the single inhibition of CK1α. Overall, these data suggest that CK1δ could have a negligible role in the regulation of autophagy in MM and that the different effects seen with D4476 might rely on different still unknown mechanisms exerted by this compound. In this particular regard, we asked if D4476 could block the autophagic flux at the lysosomal level or through the impairment of autophagic vesicles delivery to the lysosome. These data suggest the possibility that CK1 might affect lysosomal acidification. Indeed, while different CK1 isoforms interact with membrane vesicles^24,25^, and therefore they potentially could regulate the autophagosome delivery to the lysosome, our data showing a correct colocalization between the lysosomal marker LAMP-2 and LC3B in D4476-treated cells point against a D4476-mediated compromised fusion of autophagosomes and lysosomes (Fig. 4b). Thus, it remains to be elucidated if D4476 might affect the lysosomal acidification with a mechanism not already identified.

Another remarkable difference between the effects of D4476 and of CK1α and CK1α/δ silencing regards FOXO3a activation. The subcellular localization of FOXO3a and autophagic gene transcription were markedly affected by D4476 but did not change upon CK1α or CK1α/δ silencing. Indeed, in D4476 treated cells, phosphorylation of FOXO3a was reduced (Fig. 6a), FOXO3a accumulated in the nucleus (Fig. 6b, c) and the transcription of autophagic genes was initiated (Fig. S7b–d). However, in CK1α silenced cells, no nuclear accumulation of FOXO3a was observed (Fig. 6e) and a substantial pro-autophagic transcriptional program was not activated (Fig. S7e–g). Of note, the p53-dependent autophagic gene ATG4A was found upregulated in U-266 and INA-6 upon 48 h of D4476 treatment, while in H929 its expression fluctuated, being upregulated at earlier (4 h) and downregulated at longer time points (48 h). The expression of autophagic genes was previously demonstrated to be modulated by wt p53 protein in colon cancer cells^26^; among the MM cell lines analyzed, the H929 is the only one carrying wt p53. The fluctuation observed in the expression of autophagic genes in this cell line could depend by a time-dependent transcriptional activity of wt p53. Moreover, the concomitant silencing of CK1α and CK1δ did not reproduce the same effects obtained using D4476 (Fig. S9), suggesting that FOXO3a phosphorylation may not be dependent by the concomitant inhibition of CK1δ and CK1α performed by D4476, but by alternative mechanisms associated with the inhibitor.

**Fig. 7:**
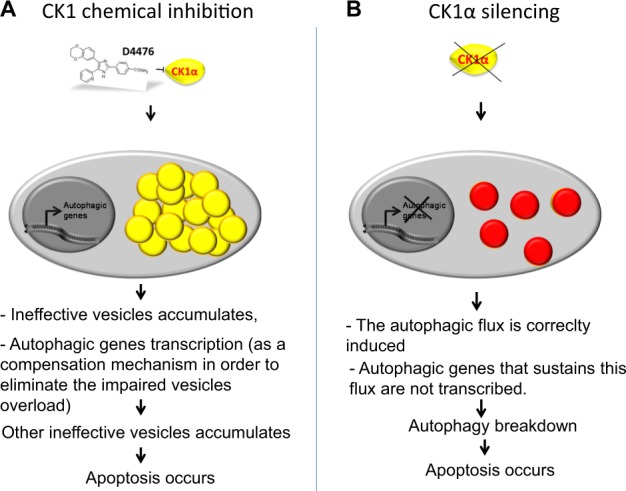
Proposed model of CK1α-mediated regulation of autophagy in MM. **a** Upon CK1α inhibition with D4476, impaired autophagic vesicles overload occurs, culminating in MM cells apoptosis; **b** in CK1α silenced cells, autophagy is triggered, but a genetic program that sustains the flux is not induced, therefore MM cells undergo autophagic breakdown culminating in cell death

In conclusion, our data confirm a central role for CK1 kinases in MM PCs biology. Our data highlight the role of CK1α and CK1δ in the regulation of autophagy. The different consequences obtained with the dual CK1α/δ inhibitor D4476 and the RNAi against CK1α or CK1δ require speculating a model whereby the two strategies of CK1 inactivation might affect autophagy and MM cell resilience to stress in different ways. In particular, in MM cells D4476 impairs the autophagic flux. Therefore, it is likely that ineffective autophagic vesicles accumulate and provoke an engulfment of the autophagic/lysosomal machinery, culminating in cell death. Nevertheless, a compensatory transcriptional autophagic genetic program is still activated (Fig. 7a). On the opposite, upon CK1α silencing, the flux is correctly triggered, however, a transcriptional program supporting autophagy is not activated. In this condition, proteins important for the autophagic machinery may be progressively depleted and since they are not resynthesized, autophagy could collapse, leading to cell death (Fig. 7b).

Our work adds further evidence supporting a role for CK1 in pro-survival cascades in MM, in particular in the stress-related autophagic pathway. MM PCs resilience to stress and survival could therefore be weakened by affecting CK1 as a novel pharmacologically targetable Achillee’s Heel.

## Materials and methods

### Patient-derived cells and cell cultures

MM cell lines (INA-6, U-266, and H929), HS-5 stromal cells and BMSC from patients were cultured as previously described^13,27,28^. The p53 and RAS mutational status is summarized in Table S1. Malignant CD138+ PCs were isolated with EasySep^TM^ kit (STEMCELL Technologies, USA) according to manufacturing procedures, after achieving informed consent according to the declaration of Helsinki. The internal Institutional ethics committee approved the use of human material (Prot. #3041 P/13). Co-cultures of patient-derived CD138+ cells and HS-5 or BMSC were performed as described in^27^ and^29^.

### Cytokines and chemicals

4-(4-(2,3-Dihydrobenzo[1,4]dioxin-6-yl)-5-pyridin-2-yl-1H-imidazol-2-yl)benzamide (D4476) was from Abcam (UK); Interleukine-6 (IL-6), chloroquine (CQ) and isopropyl-Β-D-thio-galactoside (IPTG) were from Sigma-Aldrich (Italy).

### Western blot

Total protein extraction was performed as previously described in ref. ^27^.

Nuclear and cytoplasmic proteins fractionation, was performed using buffers A and B (A: Hepes 10 mM, KCl 10 mM, EDTA 0,2 mM, DTT 1 mM, PMSF 1 mM, okadaic acids 1 μM, cocktail of phosphatase and protease inhibitors; B: Hepes 20 mM, NaCl 0,4 M, EDTA 0,2 M, DTT 1 mM, PMSF 1 mM, okadaic acids 1 μM, cocktail of phosphatase and protease inhibitors and glicerol 10% (v/v)).

Western blot (WB) was performed as previously described in ref. ^27^. The following primary antibodies were used: PARP (#9542), LC3B (2775 S), phospho FOXO3a Ser318-321 (9465 S), phospho p65 Ser536 (#3031), CK1α (2655 S) (Cell Signaling Technology, MA, USA); β-actina (A5441) (Sigma-Aldrich, Italy); GAPDH (MAB374) (Millipore, USA); p62 (#P0067), FOXO3a (ab12162), p65 (ab7970-1) (ABCAM, UK). Anti-rabbit-HRP (Cell Signaling, USA) and anti-mouse-HRP (KPL, USA) were used as secondary antibodies. Acquisition of the bands was performed in chemiluminescence using the Image Quant LAS 500 machine (GE Healthcare, USA) and the densitometric analysis of the bands was performed with the Image Quant TL software (GE Healthcare, USA).

### LC3 turnover assay

The differences in the amount of LC3-II between samples in the presence and absence of lysosomal inhibitors (i.e., CQ) represent the amount of LC3 that is delivered to lysosomes for degradation (i.e., autophagic flux)^22^. In order to evaluate the autophagic flux, cells were treated with D4476 40 μM or with CQ 40 μM or with a combination of the two compounds for 4 h. Densitometric analysis of LC3-II bands were performed. X and Y values were calculated as:

X = (densitometric value of (D4476 + CQ) sample − densitometric value of D4476 sample)

Y = (densitometric value of CQ sample − densitometric value of untreated sample)

X represents the amount of LC3 degraded upon autophagy activation; Y represents the total amount of vesicles formed upon autophagy activation.

The autophagic flux was considered activated if X > Y (consequently X−Y > 0), it was considered impaired if X < Y (consequently X−Y < 0), it was considered not affected by the treatment if X = Y (consequently X−Y is approximately 0).

LC3 turnover assay was also performed upon CK1α silencing: cells were treated with IPTG 1 mM, with CQ 40 μM or with a combination of the two chemicals for 24 h or 48 h. WB and densitometric analysis were performed as above.

### mRNA silencing

RNAi was performed through nucleofection of ds siRNA and through IPTG treatment of MM clones carrying a shRNA under the control of an IPTG-inducible promoter as in ref. ^13^.

The MM cellular clones generated were called U-266 shRNA 6044 and H929 shRNA 6044. Transduction of SCR shRNA lentiviral particles (MISSION® 3X LacO Inducible Non-Target shRNA Control Plasmid DNA, Sigma-Aldrich, Italy) was obtained in H929 (hereafter called H929 shRNA SCR clone). The MM cellular clones generated are reported in Table S2.

To induce *CSNK1A1* silencing, IPTG 0,5 mM (for U-266) or 1 mM (for H929) was added to the cell culture media and refreshed every 2 days.

### mCherry-eGFP-LC3B cellular clones generation

Retroviral particles carrying the mCherry-eGFP-LC3B plasmid (Plasmid #22418, Addgene, Cambridge, MA, USA, a gift from Jayanta Debnath) were produced according to the manufacturing procedures. Supernatant containing retroviral particles was used to transduce H929 (wt and shRNA 6044) and U-266 (wt and shRNA 6044). Transduced cells expressing mCherry-eGFP-LC3B protein were sorted using FacsAria IIIu (BD, Becton-Dickinson, Italy) and were named H929 LC3, H929 shRNA 6044 LC3 and U-266 LC3. A summary of the MM cellular clones generated is depicted in Table S2.

### Immunofluorescence

Immunofluorescence (IF) was performed as in ref. ^27^ using the following antibodies: LC3B (2775S, Cell Signaling Technology, MA, USA) and FOXO3a (ab12162, Abcam, UK). Alexa-Fluor 594-conjugated goat anti-rabbit (Life technologies, Italy) was used as secondary antibody, and specimens were mounted with Vectashield medium containing DAPI (Vector Laboratories, USA). Images were acquired with confocal microscopy Zeiss LSM 700 E90, using 63x oil objective and processed with ZEN 2012 software.

### Real-time PCR

Real-time PCR was performed as in ref. ^30^. Primers used were:

ATG4A (F: 5′′-CAGATGCTGGTTGGGGATGT-3′, R: 5′-GTTTCTCCCAGCTCCAGTCC-3’);

ATG4B (F: 5′-GCTGTCTCTGCTTGGAGGTG-3′, R: 5′-ACATCAGAAGAATCTGGACTTGG-3′);

BECN-1 (F: 5′-AGGTTGAGAAAGGCGAGACA-3′, R: 5′-GTCCACTGCTCCTCAGAGTT-3′);

CSNK1A1 (F: 5′-GGCACTGCCCGATATGCTA-3′, R: 5′-CTCGGCGACTCTGCTCAATAC-3′);

CSNK1D (F: 5′-TTCCCCGGATGCCATAACTG-3′, R: 5′-CAAGGCCCCGTACTCCAAAA-3′);

CSNK1E (F: 5′-TCAAGCCCGACAACTTCCTC-3′, R: 5′-TTTCCCGGTAGGGAATGTGC-3′);

CSNK1G (F: 5′-CAGCTGCTTTCTCGAATGGAA-3′, R: 5′-GGCTTGACATCTCGGTAAATGAG-3′);

FOXO3 (F: 5′-GGAACTTCACTGGTGCTAAG-3′, R: 5′- ACTGTCCACTTGCTGAGA-3′);

GAPDH (F: 5′-AATGGAAATCCCATCACCATCT-3′, R: 5′-CGCCCCACTTGATTTTGG-3′);

LC3B (F: 5′-CAGCGTCTCCACACCAATCT-3′, R: 5′-TCTCCTGGGAGGCATAGACC-3′);

SQSTM1/P62 (F: 5′-ATCGGAGGATCCGAGTGT-3, R: 5′-TGGCTGTGAGCTGCTCTT-3′).

### Evaluation of apoptosis

Apoptosis was assessed by Annexin V/Propidium Iodide (PI) staining (IMMUNOSTEP, Spain), as in ref. ^13^.

### Statistical analysis

Data were evaluated for their statistical significance using non parametric Student's *t*-test to evaluate if a mean value of certain distribution was significantly different from a reference value. *P*-values below 0.05 were considered statistically significant. All analyses were performed using GraphPad Prism 6 or Microsoft Excel.

## Supplementary information

Supplementary figure legends

Supplementary tables

Figure S1

Figure S2

Figure S3

Figure S4

Figure S5

Figure S6

Figure S7

Figure S8

Figure S9
