# Supplementary material for: Prosurvival autophagy is regulated by protein kinase CK1 alpha in multiple myeloma
Source: Cell Death Discov. 2019 May 21;5:98. doi: 10.1038/s41420-019-0179-1 (PMC6529432; doi:10.1038/s41420-019-0179-1)
Supplement: Supplementary file 1 — Supplementary figure legends [file 41420_2019_179_MOESM1_ESM.docx]

**SUPPLEMENTARY FIGURE LEGENDS**

**Figure S1: Quantification of CK1α, LC3B-II and p62 in CK1α silenced MM cells.**

Densitometric analysis of CK1α, LC3B-II and p62 normalized over GAPDH of **(A)** H929 transfected with a siRNA targeting *CSNK1A1* and collected after 24h-48h-72h; **(B)** H929 shRNA 6044 CK1α-silenced by the treatment with IPTG 1mM for 24h-48h-7days (7d). Densitometric values are reported as mean±SD over scramble transfected (siRNA SCR, A) or untreated (NT, B) cells (dashed lines) of at least 2 (24h), 6 (48h) and 5 (72h) independent experiments for siRNA *CSNK1A1* transfected H929 and 4 (24h), 2 (48h) and 6 (7d) independent experiments for H929 shRNA 6044. Non parametric t-test was applied. * indicates p<0.05, ** indicates p<0.01, *** indicates p<0.001, ns indicates no relevant variation compared to the control population.

**Figure S2: LC3B and p62 expression and localization in D4476 treated- MM cell lines.**

Immunofluorescence staining of LC3B **(A)** and p62 **(B)** (red) in U-266 and H929 MM cell lines treated with D4476 40μM for 48h. Nuclei are stained with DAPI (blue). For all the images 63x oil objective was used. Scale bar: 10μm.

**Figure S3: *p62/SQSTM1* mRNA transcription is enhanced in D4476-treated MM cell lines, but not in CK1α silenced MM cells.**

Histograms representing *p62* mRNA transcript level in H929 cultured in serum free media **(A)**, in H929, U-266 and INA-6 treated with D4476 40µM for different time points **(B)**, in MM cellular clones (H929 shRNA 6044 and H929 shRNA SCR) treated with 1mM IPTG for 24h-48h **(C)**, in MM (H929 and U-266) cells transfected with SCR siRNA and *CSNK1A1* directed siRNA and collected after for 24h-48h **(D)**. Data are the mean±SD of 3-9 independent experiments, are normalized over the housekeeping gene GAPDH expression and are expressed as relative percentage compared to the average of controls. Non parametric t-test was applied. * indicates p<0.05; ** indicates p<0.01; *** indicates p<0.001; ns indicates no relevant variation compared to the control population.

**Figure S4:** **The expression of other CK1 isoforms is not altered upon CK1α silencing.** Histograms represent *CSNK1A1* (CK1α), *CSNK1E* (CK1ε), *CSNK1D* (CK1δ) and *CSNK1G* (CK1γ) transcripts level in H929 transfected with siRNA SCR and siRNA *CSNK1A1* and collected after 24h-48h **(A)** and in H929 shRNA 6044 clone treated with IPTG 1mM for 24h-48h **(B).** Data are the mean±SD of 3-6 independent experiments, are normalized over the housekeeping gene *GAPDH* expression and are expressed as relative percentage compared to the average of controls. Non parametric t-test was applied. * indicates p<0.05; ** indicates p<0.01; *** indicates p<0.001; ns indicates no relevant variation compared to the control population.

**Figure S5: The autophagic flux is impaired in MM cells treated with D4476 for 48h.**

The left part of the panel shows a representative WB of LC3 turnover assay of H929 cell lines treated with the CK1 inhibitor D4476 40µM (48h), chloroquine (CQ) 40µM (4h) or the combination of the two compounds (D4476 for 48h and CQ for the last 4h). GAPDH was used as loading control. The right part of the panel shows the LC3 turnover assays, in which the densitometric quantification of LC3B-II bands was expressed as X=(D4476+CQ)-(D4476) and Y=(CQ)-(untreated cells). X>Y indicates activation of the autophagic flux (consequently X-Y>0), X<Y indicates impairment of the autophagic flux (consequently X-Y<0).

**Figure S6: Yellow ineffective autophagosomes accumulates in D4476 treated H929 LC3 cells**

H929 cells expressing mCherry-eGFP-LC3B protein (H929 LC3 clone treated with DMSO or with D4476 40μM for 18h (left panels) and 24h (right panels). Yellow fluorescence resulting by the emission of both mCherry (red) and eGFP (green) indicates a blockage of the autophagic flux. Red fluorescence resulting by the quenching of eGFP fluorescence indicates a correct induction of autophagic flux. For all the images 63x oil objective was used. Scale bar: 10μm.

**Figure S7: D4476, but not CK1α silencing, sustains an autophagic gene transcription program in MM cells.**

Histograms represent *LC3B*, *BECN-1*, *FOXO3A*, *ATG4A* and *ATG4B* transcript levels in H929 cultured in serum free media **(A)**, in H929, U-266 and INA-6 CK1 chemically inhibited with D4476 40 μM **(B, C, D)**, in H929 **(E)** and U-266 **(F)** MM cells transfected with SCR siRNA and *CSNK1A1* directed siRNA and in H929 shRNA 6044 **(G)** and H929 shRNA SCR **(H)** treated with IPTG 1mM. Cells were collected at 4h (white bars), 24h (light grey bars) or 48h (dark grey bars). Data are the mean±SD of 3-9 independent experiments, are normalized over the housekeeping gene *GAPDH* expression and are expressed as relative percentage compared to the average of controls (dashed line). Non parametric t-test was applied taking in consideration the variability of control population respect the mean (SD). + indicates p=0.05; * indicates p<0.05; ** indicates p<0.01; *** indicates p<0.001; ns indicates no relevant variation compared to control population.

**Figure S8:** **Prolonged CK1α silencing does not reduce FOXO3a phosphorylations and autophagic genes transcription.** H929 shRNA 6044 clones were treated with IPTG 1mM for 7 days. **(A)** WB analysis of CK1α, p-FOXO3a S318/321 and FOXO3a (left panel) and densitometric analysis (right panel). Densitometric values are reported as mean±SD over untreated cells (NT, dashed lines) of 9 independent experiments. GAPDH was used as loading control. **(B)** qRT-PCR of *CSNK1A1*, *LC3B* and *p62/SQSTM1* mRNA expression. Data are the mean±SD of 3 independent experiments, are normalized over *GAPDH* expression and are expressed as relative percentage compared to the average of controls. Non parametric t- test was applied. * indicates p<0.05; ** indicates p<0.01; *** indicates p<0.001; ns indicates no relevant variation compared to the control population.

**Figure S9: FOXO3a phosphorylations are not reduced and autophagic genes are not transcribed in CK1α- and CK1δ- double silenced MM cells.** H929 shRNA 6044 transfected with SCR siRNA or *CSNK1D* directed siRNA were treated with IPTG 1mM for 72h. **(A)** Representative WB of CK1δ, CK1α, pFOXO3a S318/321 and FOXO3a. GAPDH was used as loading control; **(B)** densitometric analysis of pFOXO3a S318/321 over total FOXO3a expression. Densitometric values are reported as mean±SD of 6 independent experiment; **(C)** RT-qPCR of *CSNK1D* (CK1 delta*), CSNK1A1* (CK1 alpha), *LC3B*, *p62/SQSTM1*, *FOXO3A* and *FOXO1* mRNA expression. Data are the mean±SD of 3 independent experiments, are normalized over *GAPDH* expression and are expressed as relative percentage compared to the average of controls. Non parametric t-test was applied. * indicates p<0.05; ** indicates p<0.01; *** indicates p<0.001; ns indicates no relevant variation compared to the control population.
