# Supplementary material for: Prosurvival autophagy is regulated by protein kinase CK1 alpha in multiple myeloma
Source: Cell Death Discov. 2019 May 21;5:98. doi: 10.1038/s41420-019-0179-1 (PMC6529432; doi:10.1038/s41420-019-0179-1)
Supplement: Supplementary file 2 — Supplementary tables [file 41420_2019_179_MOESM2_ESM.docx]

| **Cell line** | **p53 status** | **RAS status** |
| --- | --- | --- |
| **H929** | Wt (1) | Mut (2) |
| **U-266** | Mut (3) | Wt (2) |
| **INA-6** | Mut (1) | Mut (2) |

**Table S1: p53 and RAS status in the MM cell lines used in the study.**

| **Original cell line** | **+shRNA 6044** | **+shRNA SCR** | **+mCherry-eGFP-LC3** |
| --- | --- | --- | --- |
| **H929 wt** | H929 shRNA 6044 | H929 shRNA SCR | H929 LC3 |
| **H929 shRNA 6044** |  |  | H929 shRNA 6044 LC3 |
| **H929 shRNA SCR** |  |  | H929 shRNA SCR LC3 |
| **U-266 wt** | U-266 shRNA 6044 |  | U-266 LC3 |

**Table S2: Summary of MM cellular clones generated.**

**Supplementary References:**

1. Stühmer T, Chatterjee M, Hildebrandt M, Herrmann P, Gollasch H, Gerecke C, et al. Nongenotoxic activation of the p53 pathway as a therapeutic strategy for multiple myeloma. *Blood*. 2005; **106:** 3609-17.

2. Steinbrunn T, Stühmer T, Gattenlöhner S, Rosenwald A, Mottok A, Unzicker C, et al. Mutated RAS and constitutively activated Akt delineate distinct oncogenic pathways, which independently contribute to multiple myeloma cell survival. *Blood*. 2011; **117:** 1998-2004.

3. Liu Q, Hilsenbeck S, Gazitt Y. Arsenic trioxide-induced apoptosis in myeloma cells: p53-dependent G1 or G2/M cell cycle arrest, activation of caspase-8 or caspase-9, and synergy with APO2/TRAIL. *Blood*. 2003; **101**:4078–87.
