# Supplementary figures and images for: Prosurvival autophagy is regulated by protein kinase CK1 alpha in multiple myeloma
Source: Cell Death Discov. 2019 May 21;5:98. doi: 10.1038/s41420-019-0179-1 (PMC6529432; doi:10.1038/s41420-019-0179-1)

## Slide 1
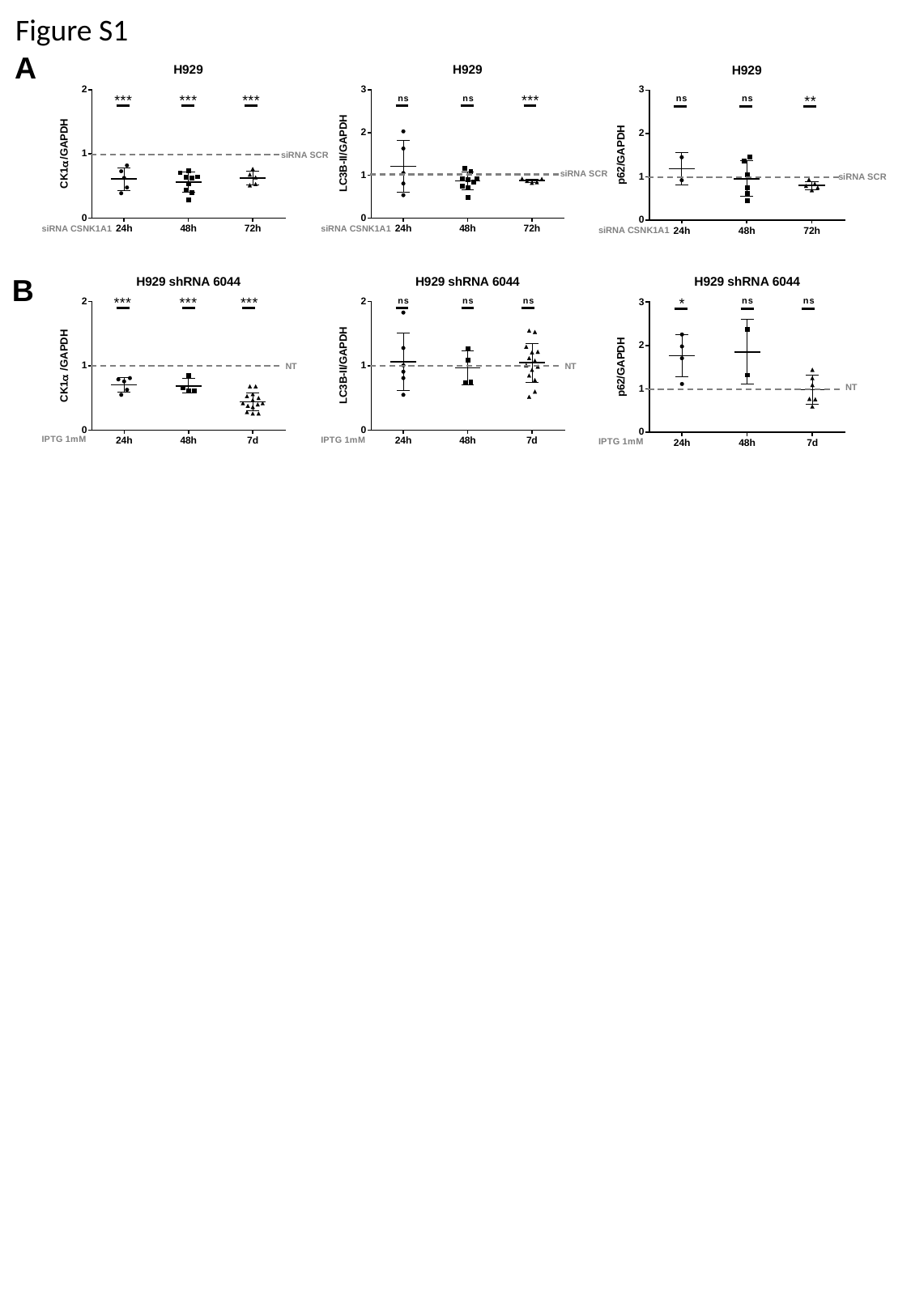

Figure S1
A
B

Supplement: Supplementary file 3 — Figure S1 [file 41420_2019_179_MOESM3_ESM.pptx]

## Slide 1
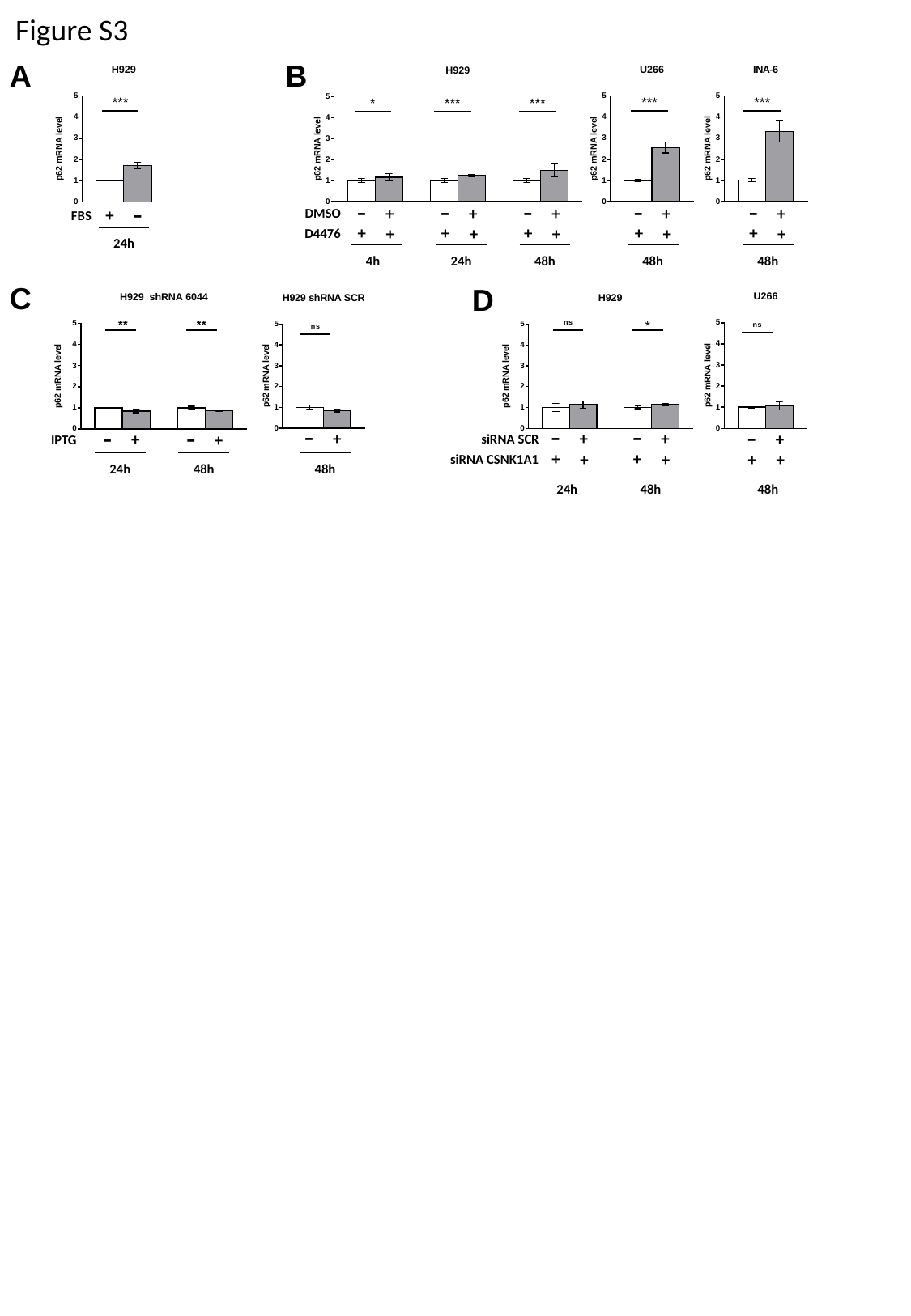

Figure S3
A
B
-
+
FBS
-
+
+
+
-
+
+
+
-
+
+
+
-
+
+
+
-
+
+
+
DMSO
D4476
24h
24h
48h
48h
4h
48h
C
D
-
+
+
+
-
+
+
+
-
+
+
+
siRNA SCR
siRNA CSNK1A1
-
+
-
+
-
+
IPTG
24h
48h
48h
24h
48h
48h

Supplement: Supplementary file 5 — Figure S3 [file 41420_2019_179_MOESM5_ESM.pptx]

## Slide 1
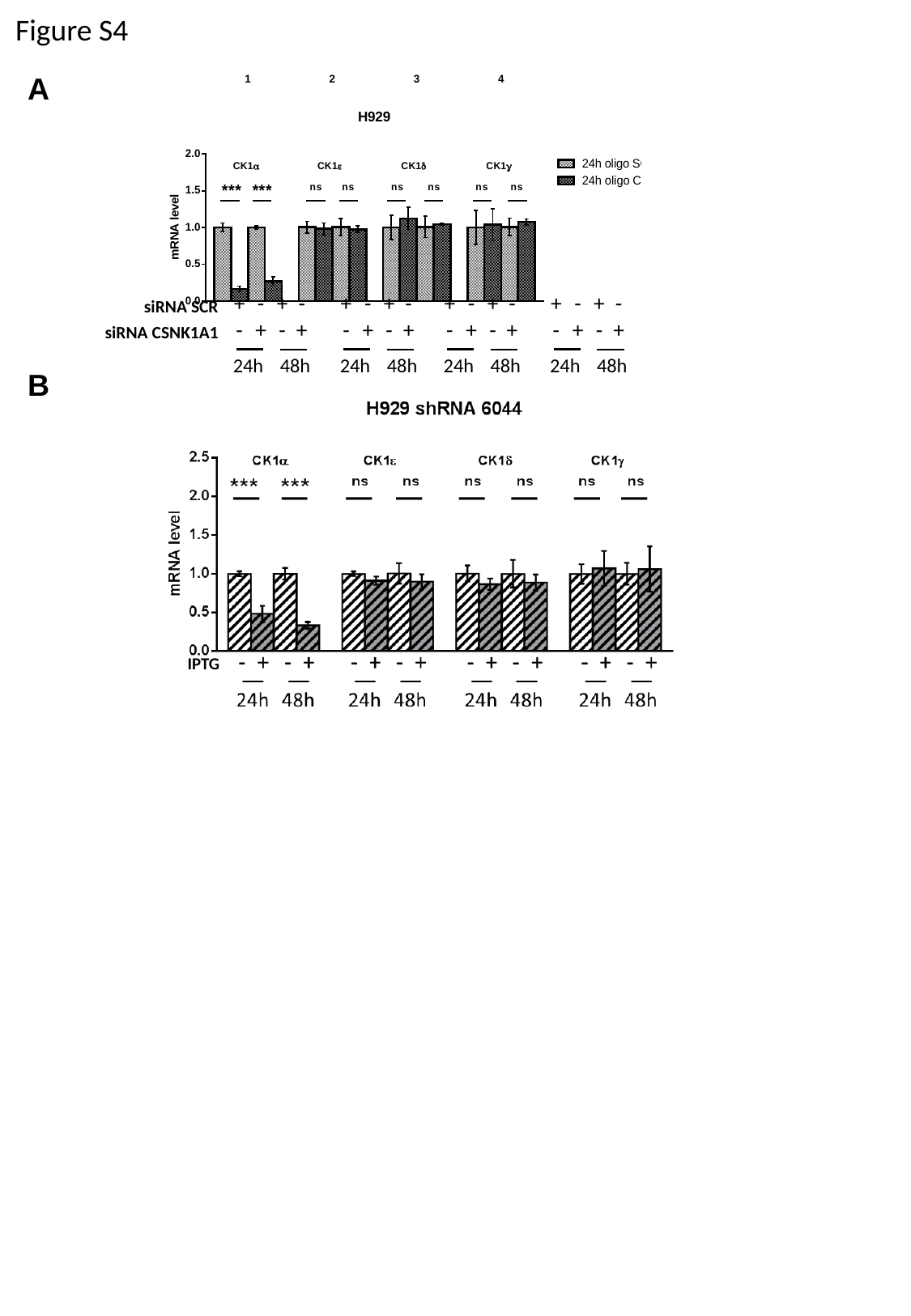

Figure S4
A
B
IPTG

Supplement: Supplementary file 6 — Figure S4 [file 41420_2019_179_MOESM6_ESM.pptx]

## Slide 1
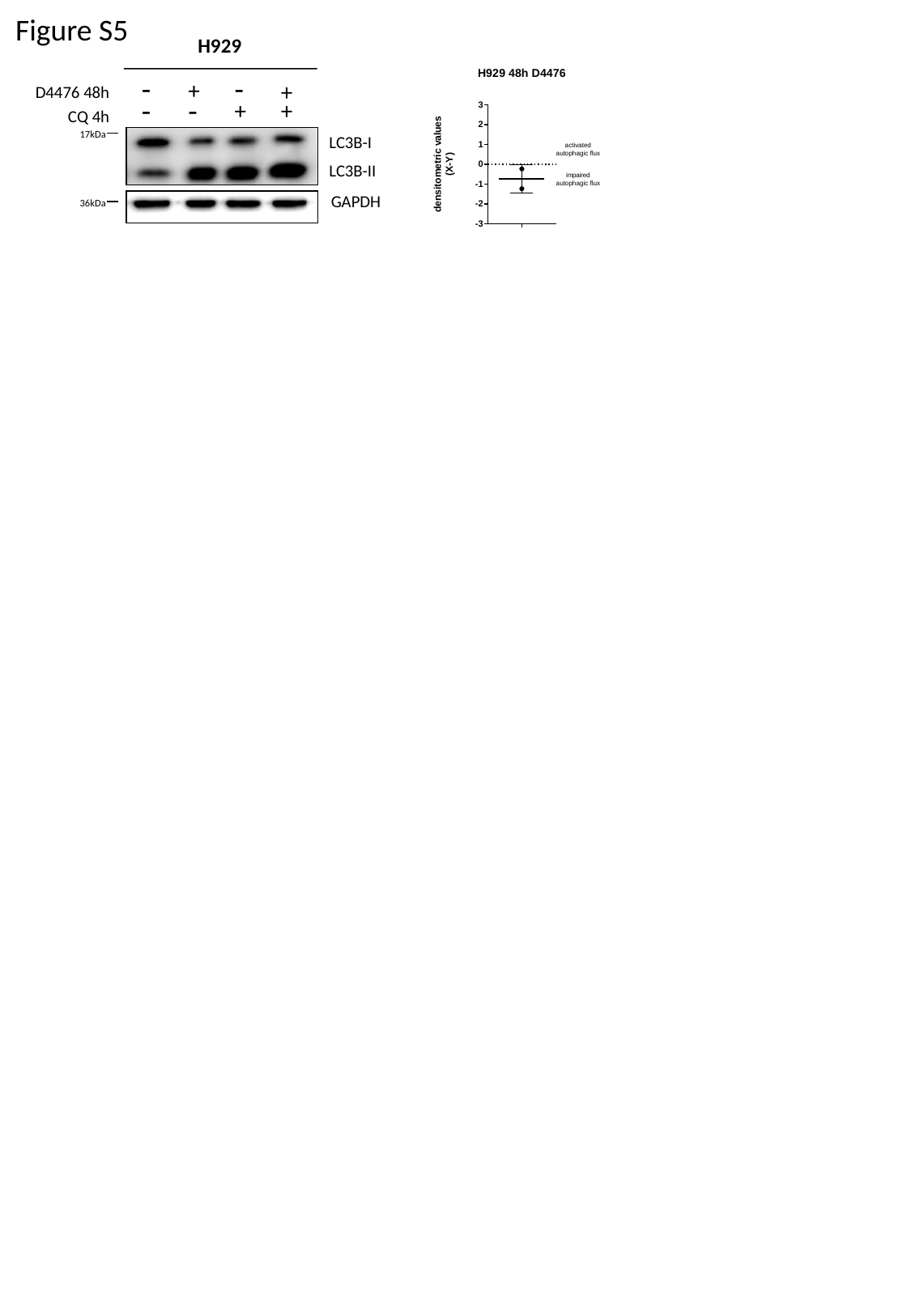

Figure S5
H929
-
-
-
+
+
-
+
+
D4476 48h
CQ 4h
17kDa
LC3B-I
LC3B-II
GAPDH
36kDa

Supplement: Supplementary file 7 — Figure S5 [file 41420_2019_179_MOESM7_ESM.pptx]

## Slide 1
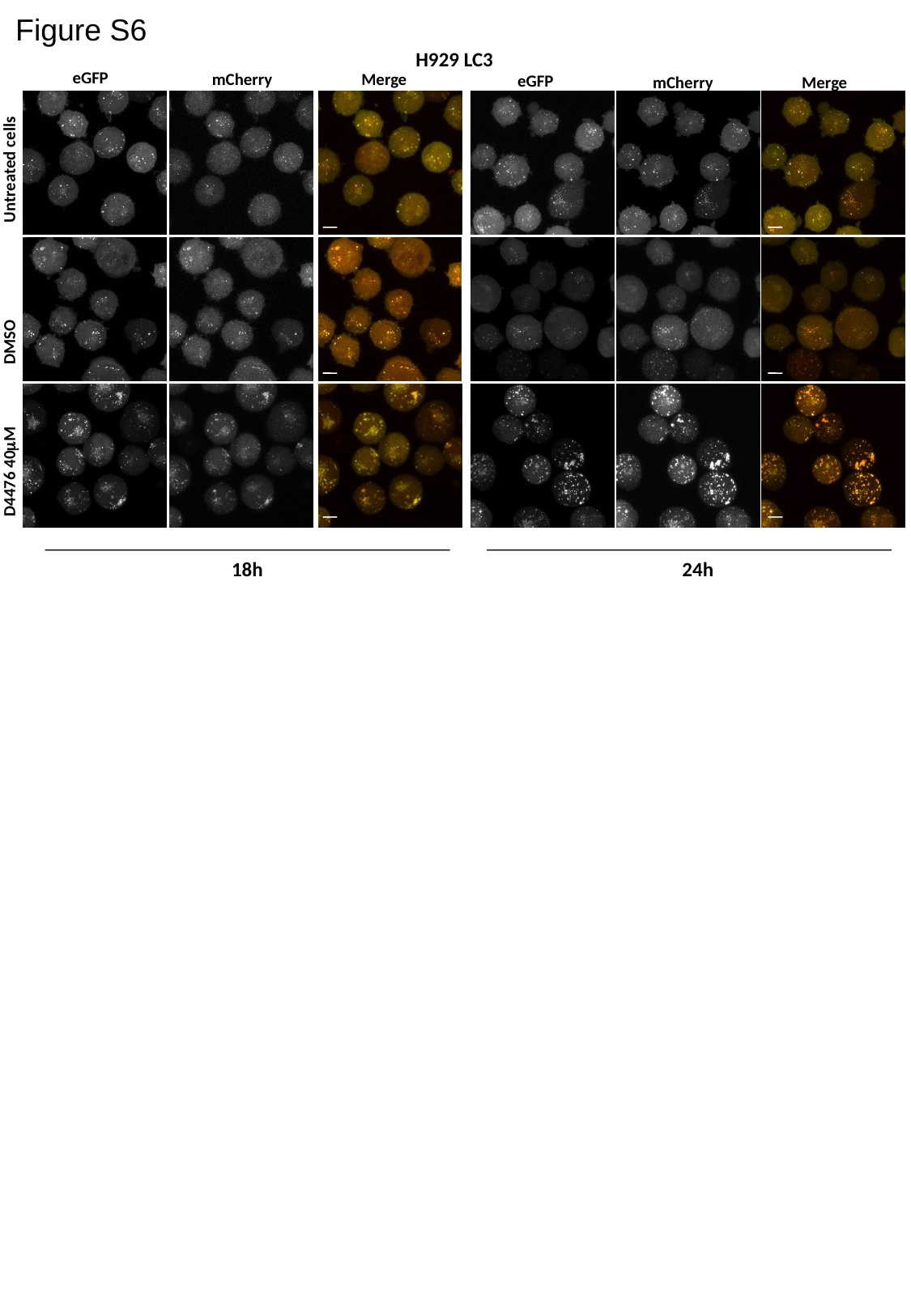

Figure S6
H929 LC3
eGFP
mCherry
Merge
eGFP
mCherry
Merge
Untreated cells
DMSO
D4476 40μM
18h
24h

Supplement: Supplementary file 8 — Figure S6 [file 41420_2019_179_MOESM8_ESM.pptx]

## Slide 1
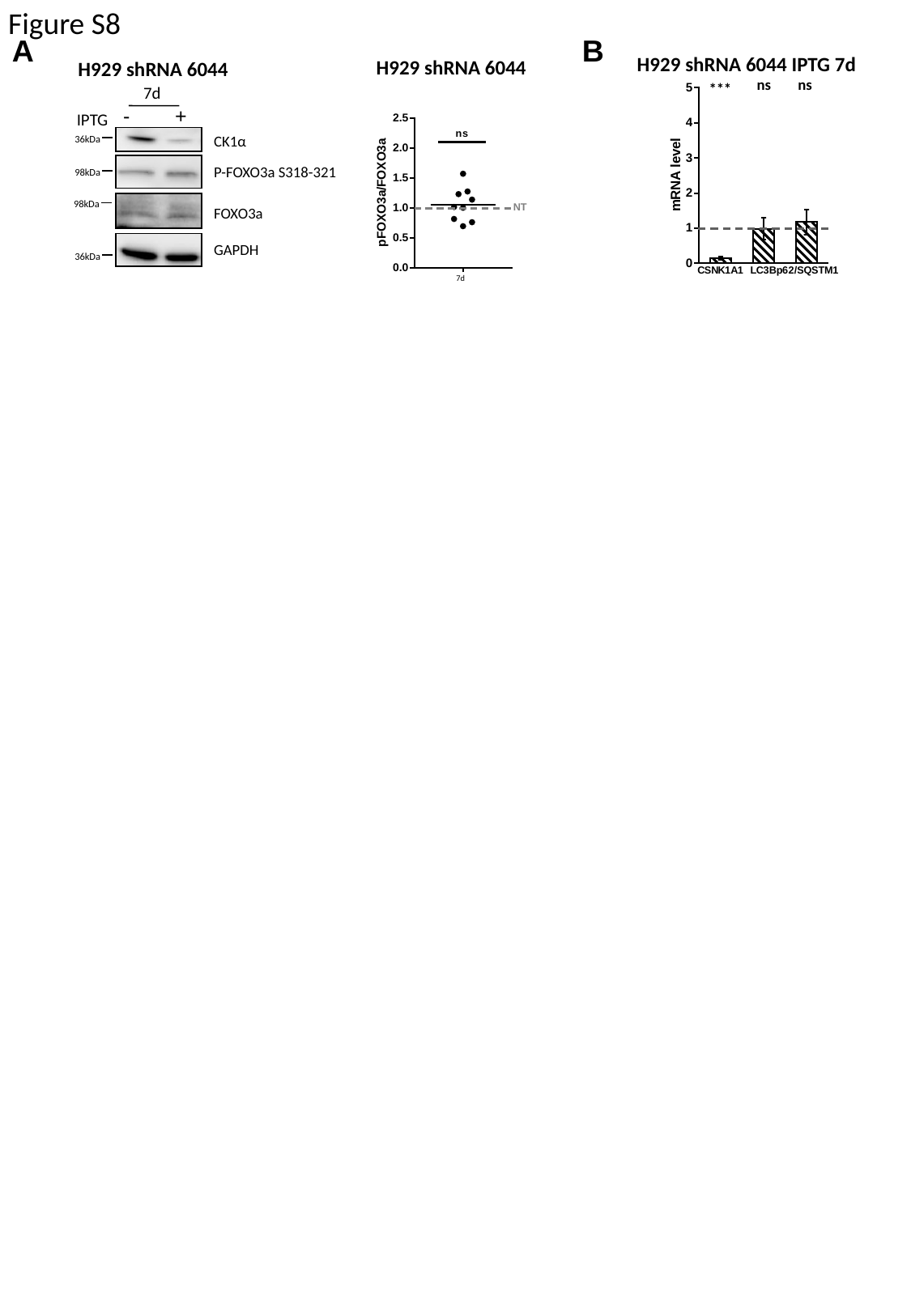

Figure S8
A
B
H929 shRNA 6044 IPTG 7d
H929 shRNA 6044
H929 shRNA 6044
7d
- +
CK1α
P-FOXO3a S318-321
FOXO3a
GAPDH
ns
ns
***
IPTG
36kDa
98kDa
98kDa
36kDa
7d

Supplement: Supplementary file 10 — Figure S8 [file 41420_2019_179_MOESM10_ESM.pptx]
