# Supplementary material for: Prosurvival autophagy is regulated by protein kinase CK1 alpha in multiple myeloma
Source: Cell Death Discov. 2019 May 21;5:98. doi: 10.1038/s41420-019-0179-1 (PMC6529432; doi:10.1038/s41420-019-0179-1)
Supplement: Supplementary file 4 — Figure S2 [file 41420_2019_179_MOESM4_ESM.pptx]

## Slide 1
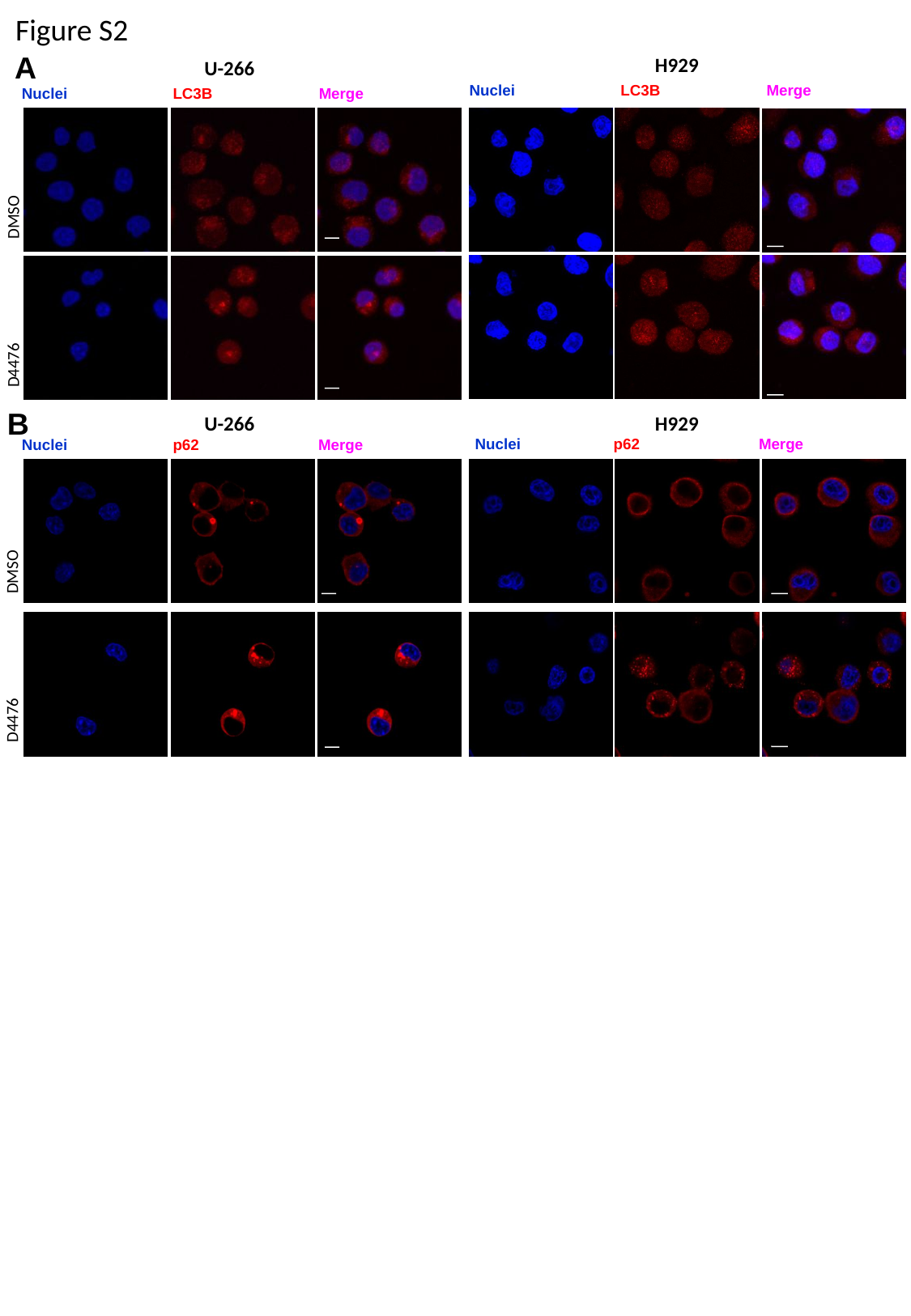

Figure S2
A
H929
U-266
Nuclei 	 LC3B Merge
Nuclei 	 LC3B Merge
DMSO
D4476
B
U-266
H929
Nuclei 	 p62 Merge
Nuclei 	 p62 Merge
DMSO
D4476
