# Supplementary material for: Prosurvival autophagy is regulated by protein kinase CK1 alpha in multiple myeloma
Source: Cell Death Discov. 2019 May 21;5:98. doi: 10.1038/s41420-019-0179-1 (PMC6529432; doi:10.1038/s41420-019-0179-1)
Supplement: Supplementary file 9 — Figure S7 [file 41420_2019_179_MOESM9_ESM.pptx]

## Slide 1
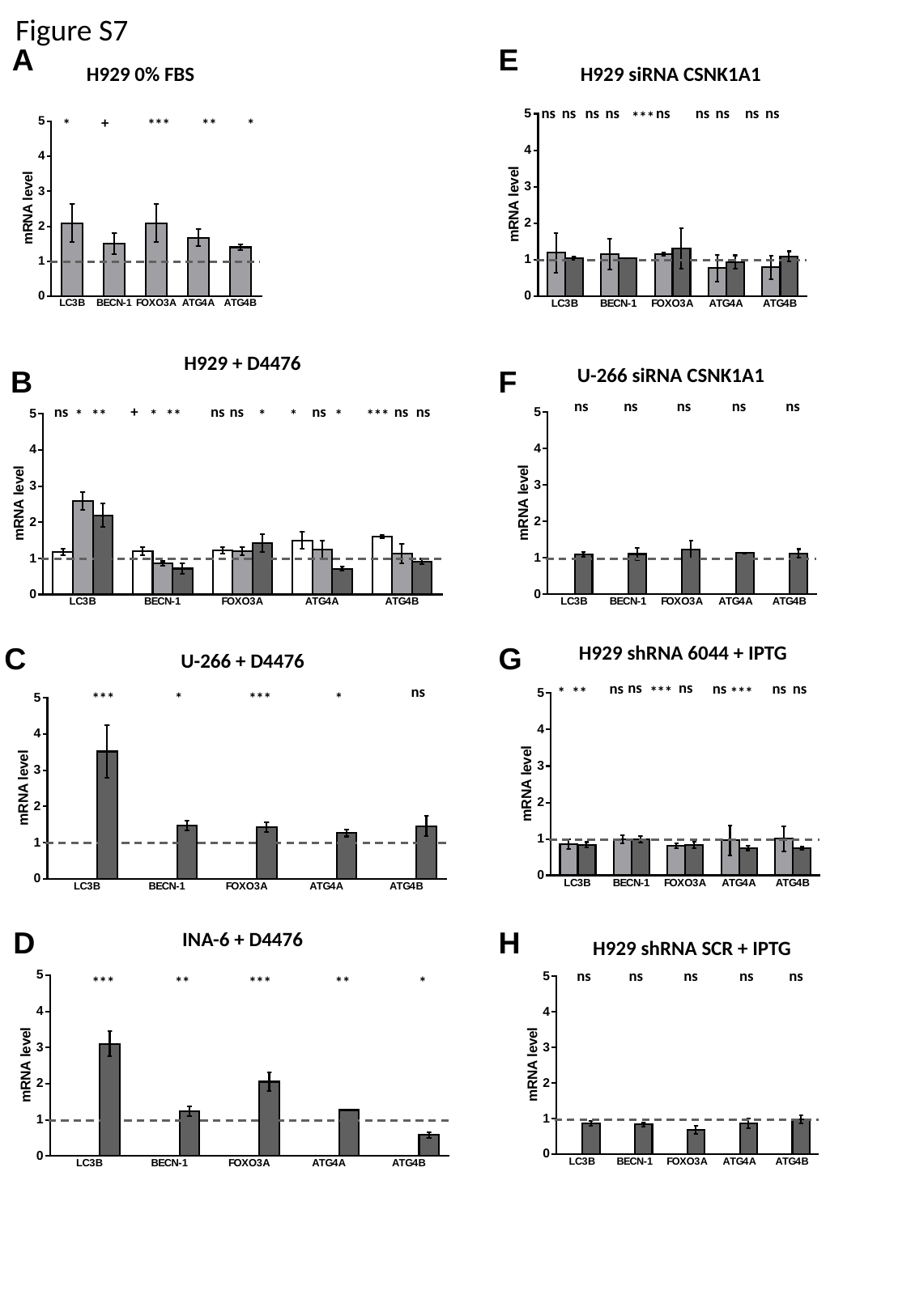

Figure S7
A
E
H929 0% FBS
H929 siRNA CSNK1A1
ns
ns
ns
ns
ns
ns
ns
ns
ns
***
+
*
***
**
*
H929 + D4476
U-266 siRNA CSNK1A1
B
F
ns
ns
ns
ns
ns
ns
+
ns
ns
ns
ns
ns
*
**
*
**
*
*
*
***
C
G
H929 shRNA 6044 + IPTG
U-266 + D4476
ns
ns
ns
ns
ns
ns
***
*
**
***
ns
***
*
***
*
D
H
INA-6 + D4476
H929 shRNA SCR + IPTG
ns
ns
ns
ns
ns
***
**
***
**
*
