# Supplementary material for: Prosurvival autophagy is regulated by protein kinase CK1 alpha in multiple myeloma
Source: Cell Death Discov. 2019 May 21;5:98. doi: 10.1038/s41420-019-0179-1 (PMC6529432; doi:10.1038/s41420-019-0179-1)
Supplement: Supplementary file 11 — Figure S9 [file 41420_2019_179_MOESM11_ESM.pptx]

## Slide 1
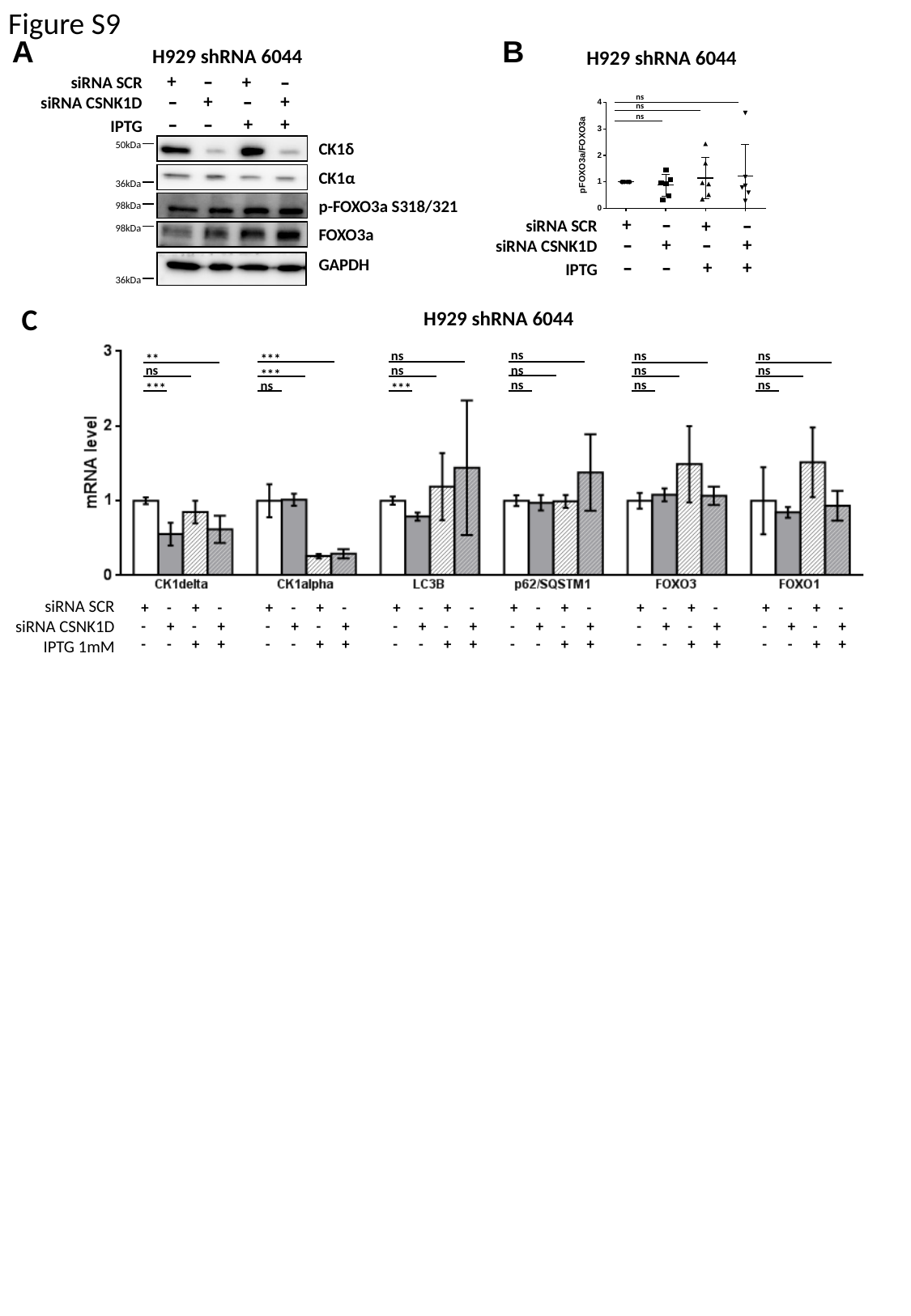

Figure S9
A
B
H929 shRNA 6044
H929 shRNA 6044
ns
ns
ns
-
-
+
+
siRNA SCR
-
-
+
+
siRNA CSNK1D
-
-
+
+
IPTG
-
-
+
+
siRNA SCR
-
-
+
+
siRNA CSNK1D
-
-
+
+
IPTG
CK1δ
50kDa
CK1α
36kDa
p-FOXO3a S318/321
98kDa
98kDa
FOXO3a
GAPDH
36kDa
C
H929 shRNA 6044
ns
ns
ns
ns
ns
***
ns
ns
ns
ns
ns
ns
***
***
ns
**
ns
***
siRNA SCR
siRNA CSNK1D
IPTG 1mM
+
-
-
-
+
-
+
-
+
-
+
+
+
-
-
-
+
-
+
-
+
-
+
+
+
-
-
-
+
-
+
-
+
-
+
+
+
-
-
-
+
-
+
-
+
-
+
+
+
-
-
-
+
-
+
-
+
-
+
+
+
-
-
-
+
-
+
-
+
-
+
+
